# Effect of postoperative enhanced recovery program care compared to conventional care following aortic valve replacement: A retrospective analysis

**DOI:** 10.1097/EA9.0000000000000118

**Published:** 2026-05-18

**Authors:** Thomas Van Bos, Charlotte Erskine, Peter Verbrugghe, Steffen Rex, Danny Feike Hoogma

**Affiliations:** From the University Hospitals Leuven, Department of Anaesthesiology, KU Leuven, Herestraat 49, B3000, Leuven, Belgium (TVB, SR, DFH), University of Glasgow, College of Medical, Veterinary and Life Sciences, Glasgow, G12 8QQ, United Kingdom (CE), University Hospitals Leuven, Department of Cardiac Surgery, KU Leuven, Herestraat 49, 3000, Leuven, Belgium (PV), University of Leuven, Biomedical Sciences Group, Department of Cardiovascular Sciences, KU Leuven, Belgium (PV, SR, DFH)

## Abstract

**BACKGROUND:**

An enhanced recovery program (ERP) after cardiac surgery is a multidisciplinary care program aimed at reducing postoperative stress response, accelerating organ function recovery, and decreasing hospital length of stay (LOS). While ERP in cardiac surgery has shown promise, high-quality evidence remains limited.

**OBJECTIVE:**

This study evaluates the impact of a postanaesthesia care unit (PACU) care (including postoperative ERP interventions) vs. conventional intensive care unit (ICU) care in patients undergoing aortic valve replacement (AVR) on hospital LOS.

**DESIGN:**

Retrospective cohort study with a Cox proportional hazards regression analysis, adjusting for BMI, EuroSCORE II and surgical access.

**SETTING:**

Single academic medical centre.

**PARTICIPANTS:**

Patients undergoing AVR with EuroSCORE II =3 and BMI <40, admitted postoperatively to either the PACU or ICU between 2011 and 2020.

**INTERVENTION:**

While pre-operative and intra-operative management were identical, postoperative care was protocolised in the PACU based on the Enhanced Recovery after Cardiac Surgery (ERAS Cardiac) pathway.

**MEASUREMENTS AND MAIN RESULTS:**

A total of 751 patients were included (345 receiving PACU care and 406 ICU care). The median hospital LOS (95% CI) was significantly shorter in the PACU care group (7 [6 to 9] vs. 9 [7 to 11] days, *P* < 0.0001) compared to ICU patients. PACU patients had a higher likelihood of early discharge [hazard ratio (HR) 1.68, 95% confidence interval (CI) 1.46 to 1.92; *P* < 0.0001] and earlier removal of catheters, tube and drains, with similar reintervention (PACU vs. ICU: 4% vs. 5%, *P* = 0.39) and mortality rates (1% in both, *P* = 0.51).

**CONCLUSION:**

Compared with conventional ICU, PACU-ERP care was associated with a significant reduction in hospital LOS in low-risk patients undergoing AVR. These findings suggest integrating the postoperative elements of ERAS Cardiac as a standard of care. Nevertheless, further prospective studies are needed for validation.

**KEY POINTS**

- In our institution, the ERAS Cardiac program is continued in the PACU, while postoperative management in the ICU follows a conventional care pathway.
- Following AVR, admission to PACU care was associated with a shorter hospital stay than the conventional ICU care pathway.
- Compared with ICU-care, PACU care in patients undergoing AVR was associated with significantly earlier removal of invasive lines and drains, as well as earlier transfer to the ward without an increase in complications.

## Introduction

In contemporary cardiac surgery, there is growing emphasis on Enhanced Recovery After Cardiac Surgery (ERAS Cardiac), originally developed for colorectal surgery. ERAS protocols have been shown to improve clinical outcomes, reduce length of stay (LOS) and enhance cost-effectiveness.^1^ As early as 1991, Engelman pioneered a fast-track care pathway in cardiac surgery, introducing a bundle of interventions aimed at reducing the incidence of common adverse events and shortening both intensive care unit (ICU) and hospital LOS.^2^ Key elements of these protocols include opioid-sparing and early extubation strategies.^3–5^ While fast-track cardiac anaesthesia is a crucial component of Enhanced Recovery Programs (ERP), ERP encompasses a broader range of interventions, including measures to optimise the pre-operative period and promote early postoperative recovery.^6,7^ Given that ERP in cardiac surgery is a relatively recent development, the current literature comprises a limited number of studies and meta-analyses.^7^

In 2011, our institution implemented a peri-operative clinical care pathway centred on the postanaesthesia care unit (PACU) as part of an ERP. Over time, additional pre-operative and intra-operative ERP interventions, now increasingly recognised as standard care, have been integrated in all patients presenting for cardiac surgery.^8^ However, postoperative interventions have been systematically implemented in PACU and rarely in ICU care. The present study retrospectively assessed the implementation and outcomes of our PACU care, compared with conventional ICU care, in patients undergoing aortic valve replacement (AVR) between 2011 and 2020. We hypothesised that PACU care, compared with conventional ICU care, was associated with a reduction in hospital LOS.

## Materials and methods

### Study design

This single-centre retrospective study (S67490) was approved by the Ethics Committee of the University Hospitals Leuven, Belgium on May 12, 2023, with a waiver of informed consent. Data were extracted from electronic health records and pseudo-anonymised prior to analysis, in accordance with the International Council of Harmonisation guidelines.

### Setting and participants

All adult patients undergoing isolated surgical AVR (via full sternotomy or minimally invasive (partial sternotomy or thoracotomy)) at University Hospitals Leuven, Belgium between 1 January 2011, and 1 January 2020, were eligible for inclusion. The inclusion criteria were a BMI <40 kg m^−2^ and a EuroSCORE II =3, consistent with the institutional standards for postoperative eligibility for the PACU care. Pre and intra-operative care were identical across both groups, while postoperative assignment to the PACU or ICU was based solely on PACU bed availability. The PACU is equipped with a limited number of beds for overnight monitoring, and when capacity was reached, patients were transferred to the ICU. Allocation was based on logistical constraints rather than clinical risk profiles.

### Postoperative care

The PACU at the authors’ institution operates under a nurse-led management protocol. Under the supervision of a staff anaesthesiologist, PACU nurses manage vasoactive drugs, administer blood products, and extubate patients once weaning criteria are met.^9^ The PACU maintains a 1 : 3 nurse-to-patient ratio, compared with a 1 : 1 or 1 : 2 ratio on the ICU. The ICU was supervised by an intensivist. Pain management also differs between units; the PACU employs a standardised multimodal analgesia protocol that excludes continuous opioid infusions, whereas the ICU lacks a uniform multimodal analgesia protocol and routinely administers continuous opioids (*e.g.*, fentanyl). Extubation, mobilisation, and catheter removal typically occur in the PACU within 1 to 6 h, 6 to 12 h and 12 to 24 h postoperatively, respectively, unless contraindicated.^9^ In contrast, ICU patients often remain sedated overnight, with extubation typically delayed for several hours with lines and drains left in place beyond ICU discharge. Ward management after discharge from either the ICU or PACU was consistent in both groups.

### Outcome variables

The hospital LOS was the primary endpoint of this study. Secondary endpoints included LOS in PACU or ICU, re-intervention rate, readmission rate, mortality and time until removal of invasive lines, tube and drains (i.e., endotracheal tube, chest drain, urinary catheter, arterial line and central venous line). Adverse outcomes were assessed up to 30 days postoperatively, and mortality was tracked for 1 year.

### Statistical methods

Baseline characteristics were compared using Student's *t*-tests for normally distributed continuous variables, Mann–Whitney *U* tests for nonparametric data and Fisher's exact test for categorical variables. The association between study group and LOS in PACU or ICU, as well as time to removal of the endotracheal tube, central venous catheter, arterial line, thoracic drain and urinary catheter, were evaluated using Cox Proportional Hazards Regression, adjusting for BMI and EuroSCORE II. A posthoc, surgical approach (minimally invasive or full sternotomy) was added as an additional covariate. Subgroup analysis was performed for patients requiring transfer to the ICU following PACU-care failure.^9^

Fine and Gray's Model was used to evaluate the relationship between study group and hospital LOS after adjusting for BMI, EuroSCORE II and surgical approach. Differences in postoperative complication rates were assessed using Poisson Regression Models. For binary outcomes, absolute risk reduction was calculated, whereas hazard ratios were used for nonbinary outcomes. An intention to treat analysis was performed for all outcomes. Patients with missing data were excluded from the dataset prior to analysis.

## Results

**Fig. 1:**
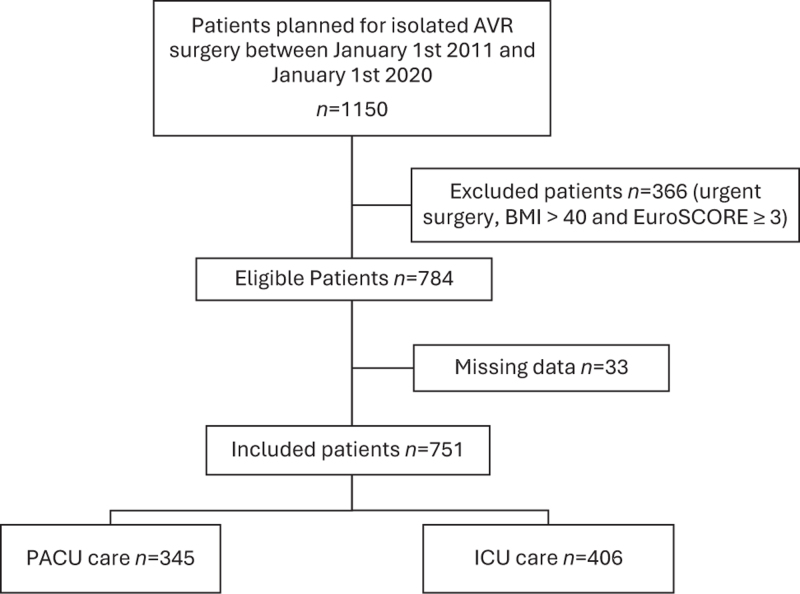
Study flowchart for patients included in the study.

AVR, aortic valve replacement; BMI, body mass index; EuroSCORE, European System for Cardiac Operative Risk Evaluation; ICU, intensive care unit; PACU, post-anaesthesia care unit.

A total of 751 patients who underwent isolated surgical AVR were included, 406 in the ICU care group and 345 in the PACU care group (Fig. 1). Baseline demographic, clinical, and procedural characteristics are summarised in Table 1. Statistically significant differences were observed for certain variables, including, EuroSCORE II, surgical approach, and duration of surgery.

**Table 1:** Baseline patient characteristics and procedural data

	All patients *n* = 751	ICU care *n* = 406	PACU care *n* = 345	*P* value
Preoperative patient data				
Age	72 [63 to 78]	72 [65 to 80]	70 [61 to 76]	<0.001
Male	447 (60)	229 (56)	218 (63)	0.063
BMI	28 ± 4.3	28 ± 4.6	28 ± 4.0	0.651
EuroSCORE II	1.3 [0.9 to 1.9]	1.5 [1.0 to 2.1]	1.1 [0.8 to 1.6]	<0.001
Ejection Fraction	60 [59 to 60]	60 [57 to 60]	60 [60 to 60]	0.243
IDDM	19 (3)	9 (2)	10 (3)	0.644
Mean pulmonary artery pressure	24 [14 to 34]	28 [14 to 36]	20 [14 to 31]	<0.001
Hypercholesteraemia	500 (67)	277 (68)	223 (65)	0.314
Serum creatinine	0.94 [0.81 to 1.08]	0.94 [0.82 to 1.08]	0.95 [0.80 to 1.07]	0.652
Dialysis	2 (<1)	2 (<1)	0 (0)	0.503
Previous cardiac procedure	21 (3)	11 (3)	10 (3)	1.000
GFR	75 ± 21.2	71 ± 17.3	80 ± 24.2	<0.001
Procedural data				
Sternotomy	332 (44)	214 (53)	118 (34)	<0.001
Minimally invasive	419 (56)	192 (47)	227 (66)	<0.001
Mini-sternotomy	405 (54)	187 (46)	218 (63)	
Mini-thoracotomy	14 (2)	5 (1)	9 (3)	
Duration of surgery (h)	3.3 ± 0.8	3.5 ± 0.8	3.1 ± 0.7	<0.001
Duration of cardiopulmonary Bypass (min)	84 [69 to 102]	83 [71 to 101]	85 [67 to 104]	0.960
Duration of aortic clamp (min)	60 [47 to 73]	59 [48 to 70]	61 [45 to 75]	0.484
Morning case	526 (70)	280 (69)	246 (71)	0.537

Data presented as median [IQR], mean ± SD or absolute incidence (% of patient group). All measures are pre-operative. *P-*value results are from comparison of means using the two-sample *t* test, Mann–Whitney *U* test and Fisher exact tests.

BMI, body mass index; EuroSCORE II, European System for Cardiac Operative Risk Evaluation; ICU, intensive care unit; IDDM, insulin dependent diabetes mellitus; GFR, glomerular filtration rate; PACU, post-anaesthesia care unit.

### Primary outcome

The overall median [IQR] hospital LOS was 8 days [6 to 10], with patients in the ICU care group staying 9 days [7 to 11] compared with 7 days [6 to 9] in the PACU care group (Table 2). Patients in the PACU had a significantly higher likelihood of early discharge (HR 1.58, 95% CI 1.38 to 1.81; *P* < 0.0001; Table 3).

**Table 2:** Observed postoperative Outcomes

	All patients *n* = 751	ICU care *n* = 406	PACU care *n* = 345	*P* value
Hospital length of stay (days)	8 [6 to 10]	9 [7 to 11]	7 [6 to 9]	<0.001
Time to ward (days)	1 [1 to 2]	2 [1 to 3]	1 [1 to 1]	<0.001
1 Year mortality	9 (1)	6 (1)	3 (1)	0.518
Time to removal of…				
Central venous catheter (days)	2 [2 to 3]	3 [2 to 5]	2 [1 to 2]	<0.001
Urinary catheter (days)	2 [1 to 3]	3 [2 to 4]	1 [1 to 2]	<0.001
Arterial line (days)	1 [1 to 2]	2 [1 to 3]	1 [1 to 1]	<0.001
Thoracic drain (days)	2 [1 to 2]	2 [2 to 2]	1 [1 to 1]	<0.001
Endotracheal tube (h)	6 [4 to 11]	10 [7 to 14]	3 [3 to 4]	<0.001
Adverse events				
30-Day readmission	9 (1)	5 (1)	4 (1)	1.000
Cardiac failure	16 (2)	13 (3)	3 (1)	0.040
Conduction failure	56 (7)	44 (11)	12 (3)	0.001
Cardiac tamponade	11 (1)	7 (2)	4 (1)	0.561
Delirium	12 (2)	10 (2)	2 (1)	0.045
Respiratory failure	12 (2)	9 (2)	3 (1)	0.242
Failure due to other cause	9 (1)	3 (1)	6 (2)	0.314
*De novo* AF	170 (23)	92 (23)	78 (23)	1.000
Know AF recurrence	23 (3)	19 (5)	4 (1)	0.005
MACCE	8 (1)	6 (1)	2 (1)	0.299
Other adverse events	5 (1)	3 (1)	2 (1)	1.000
Re-intervention				
All re-intervention	36 (5)	22 (5)	14 (4)	0.398
Due to bleeding	7 (1)	6 (1)	1 (<1)	0.132
Due to cardiac tamponade	9 (1)	5 (1)	4 (1)	1.000
Due to pacemaker insertion	17 (2)	8 (2)	9 (3)	0.627
Due to other reasons	4 (1)	3 (1)	1 (<1)	0.629

Observed postoperative data presented as median [IQR], mean ± SD or absolute incidence (% of patient group). In case of median [IQR] and mean ± SD both being listed, suggested option based on data distribution is indicated by an Asterix (*). *P*-value results are from comparison of means using the two-sample *t-*test, Mann–Whitney *U* test and Fisher exact tests.

AF, atrial fibrillation; ICU, intensive care unit; MACCE, major adverse cerebral and cerebrovascular event; PACU, post-anaesthesia care unit.

**Table 3:** Risk of postoperative outcomes across different care streams adjusted for BMI, EuroSCORE II and surgical approach

	ICU care	PACU care	Conventional ICU vs. PACU care	
	*n* = 406	*n* = 345	ARR (95% CI)	*P*-value
Individual postoperative complications:				
MACCE	6 (1)	2 (1)	<0.01 (−0.03–0.06)	0.455
Non-MACCE adverse event	3 (1)	2 (1)	<0.01 (−0.05–0.05)	0.691
Cardiac non-rhythm failure	13 (3)	3 (1)	0.02 (−0.02–0.09)	0.076
Cardiac conduction failure	44 (11)	12 (3)	0.05 (−0.04–0.15)	0.061
Cardiac tamponade	7 (2)	4 (1)	<0.01 (−0.05–0.06)	0.573
Respiratory failure	9 (2)	3 (1)	0.01 (−0.04–0.06)	0.843
Postoperative delirium	10 (2)	2 (1)	0.02 (UCL−0.09)	1.000
Other failure	3 (1)	6 (2)	−0.01 (−0.06–0.04)	0.375
*De novo* AF	92 (23)	78 (23)	0.02 (−0.13–0.17)	0.412
Recurrence of known AF	19 (5)	4 (1)	0.04 (−0.02–0.11)	0.018
30-day re-intervention	22 (5)	14 (4)	0.01 (−0.06–0.09)	0.662
Due to bleeding	6 (1)	1 (<1)	<0.01 (−0.03–0.06)	0.673
Due to tamponade	5 (1)	4 (1)	<0.01 (−0.05–0.05)	0.898
Due to pacemaker insertion	8 (2)	9 (3)	−0.01 (−0.06–0.05)	0.847
Due to other reasons	3 (1)	1 (<1)	<0.01 (−0.04–0.06)	0.480
30-day readmission	5 (1)	4 (1)	<0.01 (−0.05–0.05)	0.915
1-year mortality	6 (1)	3 (1)	<0.01 (−0.04–0.06)	0.974

			HR (95% CI)	*P*-value
Hospital length of stay (days)^b^	9 [7 to 11]	7 [6 to 9]	1.580 (1.376–1.814)	<0.001
Time to ward transfer (days)^a^	2 [1 to 3]	1 [1 to 1]	1.643 (1.393–1.938)	<0.001
Time to removal of chest drain (days)^a^	2 [2 to 2]	1 [1 to 1]	2.048 (1.742–2.406)	<0.001
Time to removal of urinary catheter (days)^a^	3 [2 to 4]	1 [1 to 2]	2.218 (1.893–2.599)	<0.001
Time to removal of central venous catheter (days)^a^	3 [2 to 5]	2 [1 to 2]	2.440 (2.070–2.878)	<0.001
Time to removal of arterial line (days)^a^	2 [1 to 3]	1 [1 to 1]	1.814 (1.529–2.152)	<0.001
Time to removal of endotracheal tube (h)^a^	10 [7 to 14]	3 [3 to 4]	8.460 (6.983–10.249)	<0.001

Results from Poisson Regression Model, unless otherwise indicated. PACU care includes all patients entered into the PACU care stream, regardless of failure. Absolute risk reduction >0 refers to a reduced risk of the event occurring in the PACU care group.

a Results from Cox proportional hazard regression models.

b Results from Fine and Grays Regression Model. Hazard ratio >1 refers to a high probability of reduced time to the given event. Bold indicates statistical significance *P* < 0.05. UCL, unable to calculate the lower confidence interval result.

AF, atrial fibrillation; ARR, absolute risk reduction; CI, confidence interval; HR, hazard ratio; ICU, intensive care unit; MACCE, major adverse cerebral or cerebrovascular events; PACU, post-anaesthesia care unit.

### Secondary outcomes

Following surgery, patients receiving conventional ICU care spent a median [IQR] of 2 days [1 to 3] in the ICU before ward transfer compared with PACU patients, who had a median [IQR] of 1 day [1 to 1] in the PACU. Assignment to PACU care was associated with a significantly shorter recovery period prior to ward transfer (*P* < 0.0001).

A total of 3% of PACU patients required transfer from the PACU to the ICU or coronary care unit, primarily due to third-degree atrioventricular block or respiratory insufficiency, while an additional 2% experienced delayed clinical deterioration necessitating escalation of care from the general ward to ICU or coronary care unit (Table S1, Supplemental Digital Content).

Regression models demonstrated that PACU care was significantly associated with earlier removal of invasive monitoring, endotracheal tube and drains. A total of 36 patients required re-intervention; however, no significant difference was observed between care pathways (Table 3). Furthermore, there was no increase in the absolute risk of individual postoperative complications in PACU care patients compared with those receiving ICU care.

## Discussion

In this retrospective study of patients with EuroSCORE II =3 requiring AVR surgery, admission to the PACU care was associated with earlier hospital discharge compared with ICU care. This effect persisted after adjusting for BMI, EuroSCORE II and surgical access. Additionally, PACU care was linked to a significantly earlier removal of invasive lines, drains and earlier discharge to the ward without an increase in complication rates.

In this era of constrained resources, strategies that enhance recovery while reducing hospital LOS are essential. The increasing demand for cardiovascular interventions continues to impose a substantial burden on patients and healthcare systems, with AVR surgeries representing a major contributor as their prevalence remains stable or is increasing in some countries.^10,11^ Consequently, the economic benefits of earlier discharge underscore the importance of ERPs, which integrate evidence-based measures across all peri-operative phases (pre, intra- and postoperative).^12^ Previous studies have demonstrated that ERP in cardiac surgery reduce hospital LOS.^13–15^ For example, Diz-Ferreira *et al.* conducted a meta-analysis evaluating ERAS protocols in cardiac surgery, reporting a mean reduction of 1.24 days without an increase in complications.^16^ However, our study specifically examined differences in early postoperative care, resulting in a more pronounced LOS reduction than that previously reported in studies assessing the entire care pathway.^17–20^ Notably, some studies failed to show any reduction in hospital LOS, despite a reduction in ICU LOS.^21,22^ We hypothesise that the reduction in hospital LOS observed in the PACU care group may be attributable to the emphasis on recovery measures (e.g., early oral intake and ambulation) and the minimisation of ‘therapeutic silence’ at the PACU. Zaouter *et al.* described ‘therapeutic silence’ as a phenomenon that occurs postextubation when early interventions (e.g., physiotherapy, oral nutrition, mobilisation, and the removal of drains and lines) are not promptly initiated.^23^ The absence of this phenomenon in our PACU group may explain the more pronounced reduction of hospital LOS. Further research is required to explore therapeutic silence after extubation and identify contributing factors, including staff training, education and other relevant variables.

Tailoring care strategies in the clinical context is essential for optimising outcomes. Serena *et al.* demonstrated that nurse-led protocols for early extubation following elective cardiac surgery may improve early extubation rates.^24^ However, these protocols have not been shown to reduce the ICU LOS.^25^ Nevertheless, nurse-led protocols may facilitate the implementation of ERAS Cardiac guidelines, although additional research is needed to confirm their downstream efficacy. The PACU in the authors’ centre follows standardised nurse-led protocols, always in co-ordination with the attending anaesthesiologist. These protocols were previously described in detail by Hendrikx *et al.*, who outlined their implementation.^9^ The ERAS Cardiac guidelines provide a Grade 1+ recommendation to extubate patients within 6 h postoperatively, when feasible.^26^ The median postoperative extubation time observed in our PACU group aligns well with this recommendation and was significantly shorter when compared with the ICU group. This difference may be partly reflected the tendency of ICU nurses to avoid weaning during night-time hours, as reported by Al Tmimi *et al.*^27^ Prolonged intubation is clinically relevant, as it can be associated with an increased risks of pulmonary complications, pneumonia and dysphagia.^28–31^ Nonetheless, we did not observe any differences in these complications, which may be attributable to the retrospective nature of this study. Prospective trials are required to further elucidate the impact of early *vs.* late extubation.^26^

Patients enrolled in the PACU care pathway experienced a significant reduction in the time required for the removal of lines and drains. The PACU protocol prioritises timely removal of central lines, arterial lines, urinary catheters, and chest drains, which is reflected in our findings. In contrast, in the ICU care group, chest drains, arterial lines and central lines were retained one day longer, and urinary catheter even two days longer. Removal of chest drains in the PACU typically occurs on postoperative day 1 to promote mobilisation and initiate both active and passive cardiopulmonary rehabilitation. The literature supports early removal of chest drains, showing no increased risk of complications such as pericardial effusion, pneumothorax, or pleural effusion.^19,22,29,30,32^ Similarly, for central line-associated bloodstream infections, one of the most effective preventive measures is the prompt removal of unnecessary catheters.^33,34^ In a study by Wei *et al.*, central line-associated bloodstream infections were observed as early as four days following central line insertion.^35^ Notably, this 4-day threshold fell within the confidence interval for the ICU group but not for the PACU group. Previous research from the National Surgical Infection Prevention Project demonstrated that prolonged postoperative use of indwelling urinary catheters, often exceeding 2 days, was associated with an increased risk of nosocomial urinary tract infections.^36^ Consistent with these findings, in our study, the median catheter duration was 2 days in the PACU group and 3 days in the ICU group.

The 1-year postoperative mortality was notably low at 1.1% and was similar between both groups. This aligns with the literature, indicating that a low EuroSCORE II reflects a low-risk patient profile.^15^ A recent Cochrane systematic review and meta-analysis concluded that fast-track protocols do not increase mortality or complication rates in patients categorised as low to moderate risk.^5,37^ Optimising resource utilisation is critical in an era of stringent cost containment. Our findings suggest that ERP implementation could provide substantial hospital benefits, including reduced hospital LOS and potential cost savings, without increasing the readmission or reintervention rates.

## Limitations

The authors acknowledge several limitations in this study. Firstly, its retrospective and single-centre design limits generalisability and precludes randomisation, introducing a potential selection bias based on the anaesthesiologist's intra-operative clinical assessment. However, because postoperative allocation was primarily determined by PACU bed availability, a degree of pseudo-randomisation was introduced. Notably, several baseline patient characteristics were significantly higher in the ICU group. To mitigate these differences and enhance comparability, statistical adjustments were made for BMI, surgical approach and EuroSCORE II. Age and GFR were not included, as both contribute to the EuroSCORE II. Secondly, the prolonged duration of surgery observed in the ICU group may be explained by a higher proportion of procedures involving sternotomy and prolonged time required for haemostasis, for which statistical adjustments were performed. Thirdly, although rare, patients experiencing severe intra-operative complications (e.g., major haemorrhage or profound haemodynamically instability), could be rerouted from PACU towards ICU if postoperative recovery was expected to be compromised. While the patients’ records were reviewed, we cannot exclude any potential effect of such cases. Fourthly, LOS variations may have been influenced by hospital bed availability and patient flow, although this probably affected only a small proportion of patients without systematic intergroup differences. Additionally, patients were managed by different nursing teams and health records were maintained in separate systems. Although these systems share fundamental similarities, a reporting bias cannot be ruled out. Finally, data collection was limited to the period before 2020, as the onset of the COVID-19 pandemic significantly affected resources and staff relocations. Future studies should consider multicentre randomised controlled trials to validate these findings and address potential biases more comprehensively.

### Strengths

Our study includes a substantial patient cohort (*n* = 751), focusing exclusively on AVR procedures to maximise consistency and minimise the variability associated with different surgical techniques. The relatively large sample size provides greater statistical power than previous studies, strengthening the validity of our findings. This design established a robust framework for evaluating ERP against a traditional care model, enabling a comprehensive analysis of outcome differences.

Additionally, since the only variable between the two groups was the postoperative phase, this study allowed for a clearer assessment of the impact of early postoperative measures on recovery without the confounding effects of differing pre-operative and intra-operative strategies.

## Conclusion

Among low-risk cardiac surgical patients undergoing AVR, continuation of ERP care in the PACU was significantly associated with reduced hospital LOS compared with conventional ICU care. This difference appears to be driven by protocolised postoperative interventions in the PACU that focus on early and active recovery. Nevertheless, large, randomised, prospective trials are necessary to confirm these findings and ensure their broader applicability. Further research focusing on pre-operative and intra-operative measures, is crucial for enhancing the overall quality of care in this patient population.

## Supplementary Material

Supplemental Digital Content
