# Supplementary material for: Effect of postoperative enhanced recovery program care compared to conventional care following aortic valve replacement: A retrospective analysis
Source: Eur J Anaesthesiol Intensive Care. 2026 May 18;5(3):1-7. doi: 10.1097/EA9.0000000000000118 (PMC13232927; doi:10.1097/EA9.0000000000000118)
Supplement: Supplemental Digital Content [file ejaic-5-e0118-s001.docx]

| ***Table S1. Baseline Patient Characteristics and Procedural Data*** | | | | |
| --- | --- | --- | --- | --- |
|  | **All Patients *N*=751** | **ICU Care**  ***n*=406** | **Conversion to ICU/CCU Care**  ***n*=10** | **Successful PACU Care**  ***n*=335** |
| **Preoperative Patient Data** | | | | |
| **Age** | 72 [63-78] | 72 [65-80] | 76 [66-80] | 70 [60-76] |
| **Male** | 447 (60) | 229 (56) | 7 (70) | 211 (63) |
| **BMI** | 28 ±4.3 | 28 ±4.6 | 28 ±2.9 | 28 ±4.1 |
| **EuroSCORE II** | 1.3 [0.9-1.9] | 1.5 [1.0-2.1] | 1.7 [1.1-2.2] | 1.0 [0.76-1.52] |
| **Ejection Fraction** | 60 [59-60] | 60 [57-60] | 60 [60-65] | 60 [60-60] |
| **IDDM** | 19 (3) | 9 (2) | 0 (0) | 10 (3) |
| **Mean Pulmonary Artery Pressure** | 24 [14-34] | 28 [14-36] | 14 [14-35] | 20 [14-31] |
| **Hypercholesteraemia** | 500 (67) | 277 (68) | 5 (50) | 218 (65) |
| **Serum Creatinine** | 0.94 [0.81-1.08] | 0.94 [0.82-1.08] | 1.02 [0.78-1.35] | 0.95 [0.80-1.07] |
| **Dialysis** | 2 (<1) | 2 (<1) | 0 (0) | 0 (0) |
| **Previous Cardiac Procedure** | 21 (3) | 11 (3) | 0 (0) | 10 (3) |
| **GFR** | 75 ±21.2 | 71 ±17.3 | 69 ±25.4 | 80 ±24.2 |
| Procedural Data | | | | |
| **Minimally Invasive** | 419 (56) | 192 (47) | 7 (70) | 220 (66) |
| **Sternotomy** | 332 (44) | 214 (53) | 3 (30) | 115 (34) |
| **Mini-Sternotomy** | 405 (54) | 187 (46) | 6 (60) | 212 (63) |
| **Mini-Thoracotomy** | 14 (2) | 5 (1) | 1 (10) | 4 (1) |
| **Duration of Surgery (Hours)** | 3.3 ±0.8 | 3.5 ±0.8 | 3.2 ±0.74 | 3.1 ±0.66 |
| **Duration of CPB (Hours)** | 84 [69-102] | 83 [71-101] | 90 [65-123] | 85 [67-104] |
| **Duration of Aortic Clamp (Hours)** | 60 [47-73] | 59 [48-70] | 59 [33-80] | 61 [45-75] |
| Data presented as median [IQR], mean ±SD or absolute incidence (% of patient group). *BMI*, Body Mass Index; *EuroSCORE II*, European System for Cardiac Operative Risk Evaluation; *IDDM*, Insulin Dependent Diabetes Mellitus; *GFR*, Glomerular Filtration Rate; *PACU*, Post-Anaesthesia Care Unit. All measures are preoperative. | | | | |
